# A Novel Organ-Specific Approach to Selectively Target Sensory Afferents Innervating the Aortic Arch

**DOI:** 10.3389/fphys.2022.841078

**Published:** 2022-03-24

**Authors:** Khalid Elsaafien, Scott W. Harden, Dominique N. Johnson, Aecha K. Kimball, Wanhui Sheng, Justin A. Smith, Karen A. Scott, Charles J. Frazier, Annette D. de Kloet, Eric G. Krause

**Affiliations:** ^1^Department of Pharmacodynamics, College of Pharmacy, University of Florida, Gainesville, FL, United States; ^2^Center for Integrative Cardiovascular and Metabolic Diseases, University of Florida, Gainesville, FL, United States; ^3^Department of Physiology and Functional Genomics, College of Medicine, University of Florida, Gainesville, FL, United States; ^4^Evelyn F. and William L. McKnight Brain Institute, University of Florida, Gainesville, FL, United States

**Keywords:** baroreceptors, sensory neurons, aortic arch, baroreflex, nodose ganglia, vagal afferents, blood pressure

## Abstract

The brain maintains cardiovascular homeostasis, in part, *via* the arterial baroreflex which senses changes in blood pressure (BP) at the level of the aortic arch. Sensory afferents innervating the aortic arch employ baroreceptors to convert stretch exerted on the arterial wall into action potentials carried by the vagus nerve to second order neurons residing within the nucleus of the solitary tract (NTS). Although the baroreflex was described more than 80 years ago, the specific molecular, structural, and functional phenotype of the baroreceptors remain uncharacterized. This is due to the lack of tools that provide the genetic and target organ specificity that is required to selectively characterize baroreceptor afferents. Here, we use a novel approach to selectively target baroreceptors. Male mice on a C57BL/6J background were anesthetized with isoflurane, intubated, and artificially ventilated. Following sternotomy, the aortic arch was exposed, and a retrograde adeno-associated virus was applied to the aortic arch to direct the expression of channelrhoropsin-2 (ChR2) and/or tdTomato (tdTom) to sensory afferents presumably functioning as baroreceptors. Consistent with the structural characteristics of arterial baroreceptors, robust tdTom expression was observed in nerve endings surrounding the aortic arch, within the fibers of the aortic depressor and vagus nerves, cell bodies of the nodose ganglia (NDG), and neural projections to the caudal NTS (cNTS). Additionally, the tdTom labeled cell bodies within the NDG also expressed mRNAs coding for the mechanically gated ion channels, PIEZO-1 and PIEZO-2. *In vitro* electrophysiology revealed that pulses of blue light evoked excitatory post-synaptic currents in a subset of neurons within the cNTS, suggesting a functional connection between the labeled aortic arch sensory afferents and second order neurons. Finally, the *in vivo* optogenetic stimulation of the cell bodies of the baroreceptor expressing afferents in the NDG produced robust depressor responses. Together, these results establish a novel approach for selectively targeting sensory neurons innervating the aortic arch. This approach may be used to investigate arterial baroreceptors structurally and functionally, and to assess their role in the etiology or reversal of cardiovascular disease.

## Introduction

The arterial baroreflex exerts moment-to-moment control and regulation over blood pressure (BP), keeping pressure within arterial walls at optimal levels for survival (Kirchheim, 1976; Brown, 1980; Kumada et al., 1990). Sensory neurons innervating the aortic arch employ baroreceptors that sense stretch in arterial walls and convey these signals to the nucleus of the solitary tract (NTS) in the hindbrain (Benarroch, 2008; Wehrwein and Joyner, 2013). These sensory afferents have their cell bodies in the nodose ganglion of the vagus nerve (NDG) and project to the NTS through the vagus nerve (cranial nerve X), providing moment-to-moment- feedback that controls heart rate (HR) and BP (Benarroch, 2008; Wehrwein and Joyner, 2013). An increase in BP results in mechanostimulation of the aortic arch wall, which activates baroreceptors (Kirchheim, 1976). This activation triggers afferent signals transmitted to the NTS. Subsequently, stimulating a neural reflex (known as the baroreflex) that reduces sympathetic, but increases parasympathetic, outflow to cardiovascular tissues restoring BP and HR to homeostatic levels (Spyer, 1989; Andresen and Kunze, 1994). While the baroreflex is well established, the molecular, structural, and functional phenotype of the sensory neurons that invoke its influence over cardiovascular function have not been completely discerned.

With the recent development of highly selective tools for probing neural circuits; viscero-sensory afferents, and neural reflexes became well characterized through targeted genetic manipulations (Saleeba et al., 2019). However, the vast majority of what’s known about baroreceptor expressing afferents arises from studies that utilize pharmacological manipulation and peripheral nerve stimulation (Schmidt, 1968; Sapru et al., 1981; Fan et al., 1996). Only recently, studies began to unravel unique markers expressed by baroreceptor containing afferents that can be utilized to characterize the baroreflex (Zeng et al., 2018; Min et al., 2019). These studies found that sensory neurons innervating the vasculature express the mechanically activated ion channels PIEZO-1 and PIEZO-2. This allowed for the specific genetic manipulation of vagal afferents expressing PIEZOs. Leading to the detailed structural and functional mapping of vagal afferents that express PIEZOs and also receive input from the aortic arch (Zeng et al., 2018; Min et al., 2019). However, vagal afferents are comprised of sensory neurons that receive a variety of input from cardio-pulmonary and gastrointestinal organs (Berthoud and Neuhuber, 2000; Kubin et al., 2006; Bai et al., 2019; Kupari et al., 2019). Furthermore, PIEZOs are not only involved in aortic stretch sensation, but also in airway (Ranade et al., 2014; Woo et al., 2015; Nonomura et al., 2017), and stomach stretch sensation (Alcaino et al., 2017; Min et al., 2021; Oh et al., 2021). Thus, there is a need to develop approaches that target single modality sensory vagal afferents that innervate the aortic arch. Such tools would allow the specific/selective structural and functional characterization of baroreceptor expressing afferents.

In the current study, we sought to develop a novel organ-specific approach to selectively target sensory neurons innervating the aortic arch. Using a retrograde adeno-associated virus (AAVrg) we directed the expression of channelrhodopsin-2 (ChR2) and/or tdTomato (tdTom) within neurons that presumably function as baroreceptors innervating the aortic arch. Our anatomical analysis revealed the successful expression of tdTom selectively in these neurons. Consistent with the established neuroanatomical circuit of the baroreflex, we observed expression of tdTom in nerve endings innervating the aortic arch that projected through the aortic depressor nerve (ADN) to the NDG of vagus nerve. The cell bodies of these sensory afferents that reside in the NDG were found to express PIEZOs, and they send projections that terminate in the NTS. Our *in vitro* electrophysiology confirmed a functional glutamatergic connection between the baroreceptors, targeted by our approach, and neurons of the NTS. Furthermore, *in vivo* optogenetic stimulation of the cell bodies of neurons that express baroreceptors produced depressor responses. Altogether, our results demonstrate that our organ-specific approach to selectively target sensory neurons innervating the aortic arch is anatomically and functionally valid.

## Materials and Methods

### Animals

Studies were conducted in 23 male C57BL/6J mice. Mice were 8–10 weeks old at the initiation of studies. All animals were maintained in temperature and humidity-controlled rooms on 12:12-h light-dark cycles. In all cases, food and water were available *ad libitum*. All procedures were approved by the Institutional Animal Care and Use Committees at the University of Florida and were conducted in accordance with the National Institutes of Health Guide for the Care and Use of Laboratory Animals.

### Viral Constructs

Retrograde Adeno-Associated Viral vectors [AAVrg; i.e., AAV-CAG-hChR2-H134R-tdTomato (Cat#: 28017-AAVrg) and pAAV-CAG-tdTomato (codon diversified) (Cat#: 59462-AAVrg)] that allow the expression of tdTomato (tdTom) and/or the light sensitive cation channel, channelrhodopsin-2 (ChR2), were obtained from Addgene, and used at a titer of ≥7 × 10^12 vg/mL.

### Cardiothoracic Surgery

Animals were anesthetized using isoflurane (3–5% in O_2_) and administered analgesics (Bupernex; 0.1 mg kg^–1^ s.c.). Following the induction of anesthesia, animals were intubated and artificially ventilated (Tidal Vol; 0.2 ml, Min Vol; 26 ml min^–1^, Pressure 21 cmH_2_O). A longitudinal midline cervical incision was made from the sternal notch to the mid-chest. Following the retraction of the pre-tracheal muscles, smooth-tipped micro-surgical forceps were inserted behind the sternum and over the trachea to bluntly dissect the pleura away. A bone nipper was then used to perform a midline sternotomy (approximately 3–4 mm). This exposed the aortic arch in the pericardial cavity. Subsequently, a glass micropipette was then filled with the viral constructs. Approximately 1 μl was applied to the aortic arch by “painting” the virus on the vascular wall. Briefly, viral constructs were applied to the vascular wall of the aortic arch where the right brachiocephalic trunk, left common carotid, and left subclavian artery branch off the aortic arch. Viral constructs were left *in situ* for 5 min to allow for the diffusion of AAVrg into the aortic arch. It is worth noting that the viral constructs were not injected into the aortic arch or systemic circulation, but rather, were discretely painted onto the vascular wall. Finally, the sternum and the skin were sutured with 5–0 absorbable and non-absorbable sutures, respectively. Mice were allowed to recover for 21 days before neuroanatomical, *in vitro* electrophysiological, or *in vivo* cardiovascular studies were performed.

### Experimental Protocols

#### Neuroanatomical Studies

Mice that received an AAVrg to direct the expression of tdTom (*n* = 4) within the sensory neurons that innervate the aortic arch were anesthetized with pentobarbital (50 mg kg^–1^, i.p.) and transcardially perfused with RNase-free-isotonic saline followed by 4% paraformaldehyde. The aortic arch and the carotid sinus were collected and stored in RNase-free saline at 4°C for further processing. The left and right nodose ganglia (LNG and RNG, respectively) were dissected and placed in 4% paraformaldehyde for 5 min before they were stored in 20% RNase-free sucrose at 4°C. Brains were extracted and post-fixed for approximately 4 h after which they were stored in 30% RNase-free sucrose at 4°C.

The intact aortic arch, carotid sinus, LNG, and RNG were mounted (whole-mount) onto Fisherbrand Superfrost Plus Gold Microscope Slides (Thermo Fisher Scientific, Waltham, MA, United States). A laser-scanning confocal microscope (Nikon Instruments Inc., Melville, NY, United States) was used to assess viral expression at the aortic arch and the NDG. Tissue from animals with successful expression of the viral constructs were further processed. The LNG and RNG were processed for RNAscope *in situ* hybridization, and the hindbrains were processed for immunohistochemistry.

#### *In vitro* Electrophysiology

##### Slice Preparation

Mice that received an AAVrg to direct the expression of ChR2 and tdTom (*n* = 7) within baroreceptor expressing neurons were anesthetized with ketamine HCl (0.1 mL of 100 mg mL^–1^; i.p.). Following decapitation and rapid brain extraction, a Leica VT1000 vibratome was used to obtain 300 μm thick horizontal sections submerged in ice-cold sucrose-laden artificial cerebrospinal fluid (aCSF) containing (in mM): 205 sucrose, 10 dextrose, 1 MgSO_4_, 2 KCl, 1.25 NaH_2_PO_4_, 1 CaCl_2_, and 25 NaHCO_3_, oxygenated with 95% O_2_/5% CO_2_. Slices were transferred to an incubation chamber containing low-calcium high-magnesium aCSF (designed to minimize excitotoxicity) containing (in mM): 124 NaCl, 10 dextrose, 3 MgSO_4_, 2.5 KCl, 1.23 NaH_2_PO_4_, 1 CaCl_2_, and 25 NaHCO_3_, oxygenated with 95% O_2_/5% CO_2_ and maintained at 35°C. After 30 min the incubation chamber was removed from heat and permitted to passively equilibrate to room temperature for at least 30 min prior to recording. Recordings were performed in aCSF containing (in mM): 126 NaCl, 11 dextrose, 1.5 MgSO_4_, 3 KCl, 1.2 NaH_2_PO_4_, 2.4 CaCl_2_, and 25 NaHCO_3_, oxygenated with 95% O_2_/5% CO_2_ and maintained at 28°C flowing through a perfusion chamber (JG-23W/HP, Warner Instruments, Holliston, MA, United States) at a rate of 2 mL min^–1^. Patch pipettes were prepared from 1.5 mm OD/0.86 mm ID filamented fire-polished borosilicate glass (Sutter Instruments BF150-86-10, Novato, CA, United States) using a Flaming/Brown pipette puller (Sutter Instrument SU-P97, Novato, CA, United States) to achieve a 4–6 MΩ open-tip resistance when filled with a cesium-based internal solution containing (in mM): 115 Cs-gluconate, 10 di-tris phosphocreatine, 10 HEPES, 0.5 EGTA, 2 MgCl_2_, 4 Na_2_ATP, 0.4 Na_3_GTP, 5 QX-314 chloride, adjusted to pH 7.25 and 295 mOsm. The liquid junction potential of this pipette solution against aCSF is calculated to be 15.95 mV, and data presented are uncorrected. Cells were visualized with an Olympus BX51WI upright stereomicroscope using IR-DIC optics and a 12-bit CCD camera (QImaging Rolera-XR) and μManager software (Edelstein et al., 2010). Fluorescence illumination (for imaging and optogenetic stimulation) was achieved using a TTL-controlled LED light source (X-Cite 110LED, Excelitas Technologies, Waltham, MA, United States) and standard filter cubes (XF404, XF414, from Omega Optical).

##### Patch-Clamp Electrophysiology

Electrophysiological recordings were performed using a CV-7B headstage with a MultiClamp 700B amplifier, a DigiData 1440A digital acquisition system, and pClamp 11 software (Axon Instruments/Molecular Devices). Recordings were acquired at 20 kHz and low-pass filtered using a 4-pole Bessel filter with a 2 kHz –3 dB cutoff. Passive membrane properties for each neuron were determined using a voltage-clamp protocol containing a 10 mV hyperpolarizing step (to measure membrane and access resistance) and a pair of 10 mV ramps (to measure whole-cell capacitance) (Golowasch et al., 2009). Optogenetic excitation was achieved using a 20 ms pulse of blue light delivered through the objective lens. Membrane tests (–10 mV hyperpolarizing steps) were repeated in voltage clamp every 10 s during time-course experiments and cells were omitted if access resistance changed by more than 10 MΩ over the course of an experiment. All experiments were performed in the continuous presence of the GABA_A_ receptor antagonist picrotoxin (PTX, 100 μM). In experiments where light-evoked currents were challenged with glutamate receptor antagonists, the extracellular solution was switched to aCSF containing DNQX (20 μM) to block AMPA and kainate receptors. In cases where light-evoked currents were still visible NMDA receptor antagonist AP5 (40 μM) was also added to the aCSF. To quantify optogenetic excitation, data were low-pass filtered using a Gaussian filter (1 ms halfwidth), light-evoked currents were quantified relative to baseline as the negative peak observed in a 40 ms window following light exposure.

#### *In vivo* Cardiovascular Recordings

Mice that received an AAVrg to direct the expression of ChR2 and/or tdTom (*n* = 6 per group) within baroreceptor expressing afferents were anesthetized with isoflurane (3–5% in O_2_). A Millar catheter (Model SPR1000, Millar, Inc., Houston, TX, United States) was implanted into the left common carotid artery as previously described (Elsaafien et al., 2021; Frazier et al., 2021; Mohammed et al., 2021). Fiberoptic posts where then placed above the LNG. Following establishing baseline BP, blue light was illuminated in the LNG (473 nm, 10 mW output; 20 ms pulse width; 1, 5, 10 Hz for 1 min). Blood pressure (BP) and heart rate (HR) data were sampled/recorded at 1 kHz and analyzed using LabChart 8 software (AD Instruments, Colorado Springs, CO, United States). BP and HR were analyzed at 30 s bins for 2 and 5 min prior to and following optical stimulation, respectively.

### RNAscope *in situ* Hybridization and Immunohistochemistry

#### Section Preparation

Each NDG was sectioned at 10 μm into 6 serial sections using a Leica CM3050 S cryostat (Leica, Buffalo Grove, IL, United States). Sections were immediately mounted onto Fisherbrand SuperFrost Plus Gold Microscope Slides (Thermo Fisher Scientific, Waltham, MA, United States). After air-drying at room temperature for 1 h, slides were rinsed with PBS, incubated in 4% PFA for 5 min, and then dehydrated for 5 min in 50, 75, and 100% EtOH. Following that, slides are incubated in H_2_O_2_ for 10 min, rinsed with PBS, and allowed to dry for 10–15 min before being stored at –80°C for further processing. The hindbrain was sectioned at 30 μm into 4 serial coronal sections using a Leica CM3050 S cryostat (Leica, Buffalo Grove, IL, United States). Sections were stored in cryoprotective solution at –20°C, until further processing.

#### *In situ* Hybridization

RNAscope *in situ* hybridization was performed using the RNAscope^®^ V2 Multiplex Fluorescent Reagent Kit (Advanced Cell Diagnostics, Newark, CA, United States) as per the manufacturer’s instructions with slight modification to the pretreatment procedure that allows for preservation of the tdTomato, while still providing optimal mRNA signal. The probes used for these studies were as follows: PIEZO-1 (Mm-Piezo1-O2; Cat. No. 529091), and PIEZO-2 (Mm-Piezo2-E39-E44; Cat. No. 433421). Upon completion of the hybridization for either PIEZO-1 or PIEZ0-2, sections immediately underwent IHC for m-Cherry to amplify the tdTom signal.

#### Immunohistochemistry

Following RNAscope *in situ* hybridization, standard IHC protocols were used to amplify the tdTom signal in sections through the NDG. Sections were incubated in rabbit anti-m-Cherry at 1:2000 (Cat#: ab167453, abcam). Secondary antibodies were purchased from Jackson ImmunoResearch (Cy3 donkey anti-rabbit, Cat#: 711-165-152) and used at a 1:500 dilution. Briefly, sections were rinsed then incubated in blocking solution (2% normal donkey serum and 0.2% Triton X in 50 mM KPBS) for 2 h at 25°C and then in the primary antibody (diluted in blocking solution) for 18 h at 4°C. Sections were again rinsed 5 × 5 min (50 mM KPBS) before incubation in the secondary antibody in blocking solution for 2 h at 25°C. After a final series of rinses, slides were allowed to air dry and then coverslipped using Prolong™ Gold Antifade Mountant.

### Image Capture and Processing

Images were captured and processed using a laser-scanning confocal microscope (Nikon Instruments Inc., Melville, NY, United States). Large coronal scans captured at 10 x magnification throughout the aortic arch and carotid sinus (whole-mount), NDG (whole-mount LNG and RNG), and hindbrain (coronal sections) were acquired to assess the expression of tdTomato. Regions with high expression of tdTom were then imaged at 20x magnification and z-stacks through sections were acquired. For *in situ* hybridization in the NDG, z-stacks of the proteins and mRNAs of interest were captured at 40° magnification. An average of 20 optical sections were collected per z-stack (0.5 μm between z-steps). Sections hybridized with the probes of interest (PIEZO-1 or PIEZO-2) were used to determine the exposure time and image processing required to provide optimal visualization of RNA signal. As described in detail in de Kloet et al. (2016), these same parameters were then used to assess background fluorescence in sections hybridized with the negative control probe (DapB). Importantly, using these exposure times and image processing parameters there was minimal or no fluorescence in sections hybridized with the negative control probe. All representative photomicrographs were then prepared using Adobe Photoshop 2021 to adjust brightness and contrast to provide optimal visualization. Final figures and schematics were prepared in Adobe Illustrator 2021.

### Statistics

All *in vitro* electrophysiology data were analyzed using a custom software written in OriginC by CJF (Originlab, Northampton, MA, United States). The effect of voltage or glutamate receptor antagonists on light-evoked currents across cells was evaluated using ratio *t*-tests (paired *t*-tests performed on log-transformed peak currents). Data in the text are reported as mean ± SEM.

All *in vivo* cardiovascular data were analyzed in Labchart 8 (AD Instruments, Colorado Springs, CO, United States). Two-way ANOVAs with Tukey’s multiple comparison tests were performed using GraphPad Prism. Data are expressed as mean ± SEM.

## Results

### Sensory Neurons Innervating the Aortic Arch Terminate in the Caudal Nucleus of the Solitary Tract

**FIGURE 1:**
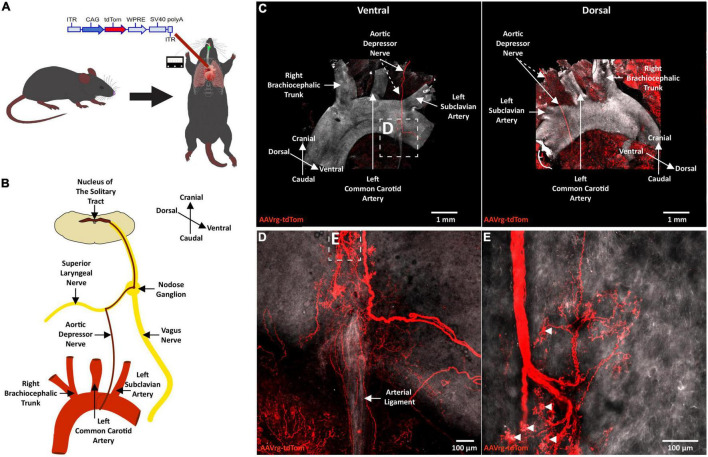
Terminals of sensory neurons innervating the aortic arch are selectively labeled with a retrograde adeno-associated viral vector. **(A)** A schematic diagram depicting the cardiothoracic surgery performed to allow for the application of an AAVrg to the aortic arch to direct the expression of tdTom within the sensory neurons innervating the aortic arch (*n* = 4). **(B)** A schematic demonstrating the sensory innervation of the aortic arch. **(C)** A representative photomicrograph of a whole-mount aortic arch showing selective expression of tdTom in the sensory nerve terminals innervating the aortic arch ventrally (left) and dorsally (right). **(D,E)** Higher magnification scans revealing the morphology of the sensory nerve terminals innervating the aortic arch.

**FIGURE 2:**
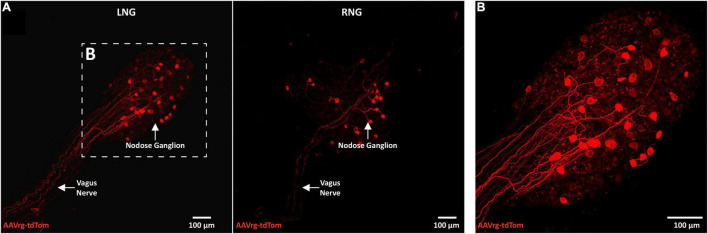
The cell bodies of sensory neurons innervating the aortic arch are in the nodose ganglia of the vagus. **(A)** A representative photomicrograph of a whole-mount left nodose ganglion (LNG) and right nodose ganglion (RNG) depicting the expression of tdTom within fibers in the vagus nerve and cell bodies of the nodose ganglia (*n* = 4). **(B)** A higher magnification of cell bodies expressing tdTom in the LNG.

**FIGURE 3:**
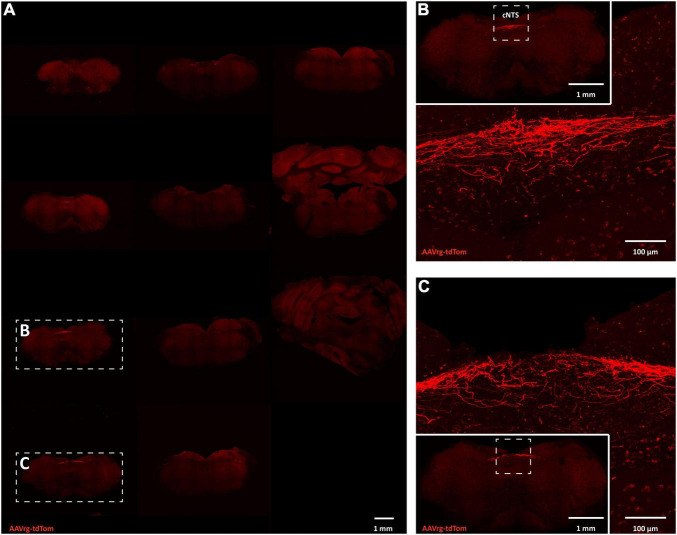
Sensory neurons innervating the aortic arch terminate in the nucleus of the solitary tract. **(A)** Large scans of coronal sections of hindbrain revealing expression of tdTom within fibers in the caudal nucleus of the solitary tract (cNTS; *n* = 4). **(B,C)** Higher magnification representative image of the cNTS.

Neuroanatomical studies were performed to assess the specificity and selectivity of the current approach in targeting the sensory neurons that innervate the aortic arch. Mice received an AAVrg to direct the expression of tdTom (*n* = 4) within the nerve endings innervating the aortic arch (Figure 1A). After 3 weeks, the aortic arch, NDG and the hindbrain were collected. Imaging of whole-mount samples revealed the incoming ADN as it innervates the aortic arch. The ADN contacted the peak of the left common carotid and the left subclavian arteries and bifurcated caudally with each branch innervating the dorsal or ventral surface in a saddle-like pattern (Figure 1B). tdTom was observed within fibers surrounding the aortic arch and projecting through the ADN (Figure 1C). Higher magnification scans revealed the morphology of the nerve endings as abundant flower-spray endings (Figures 1D,E). This is consistent with previous description of the sensory innervation of the aortic arch (Aumonier, 1972; Krauhs, 1979; Min et al., 2019). These sensory afferents ascend through the ADN to join the vagus nerve through the superior laryngeal nerve (Figure 1B). To assess the specificity of our approach in targeting the aortic arch, the carotid sinus was imaged. No fibers or nerve endings were observed at the carotid bifurcation (Supplementary Figure 1). The NDG of the vagus nerve is where the cell bodies of these sensory neurons reside (Aumonier, 1972; Krauhs, 1979; Zeng et al., 2018; Min et al., 2019). We observed the expression of tdTom in fibers running through the vagus nerve and in cell bodies within LNG and RNG (87 ± 15 neurons; Figure 2). From the NDG of the vagus, these sensory neurons project to and terminate in the NTS (Mendelowitz et al., 1992). By scanning coronal sections of the whole extent of the hindbrain, we observed that terminals expressing tdTom were only localized to the cNTS (Figure 3). Our neuroanatomical results are consistent with previous reports describing the neuroanatomy of sensory neurons that innervate the aortic arch. Hence, our approach to retrogradely label nerve endings innervating the aortic arch is highly specific and selective in targeting single modality fibers.

### Cell Bodies of the Sensory Neurons Innervating the Aortic Arch Express PIEZOs

**FIGURE 4:**
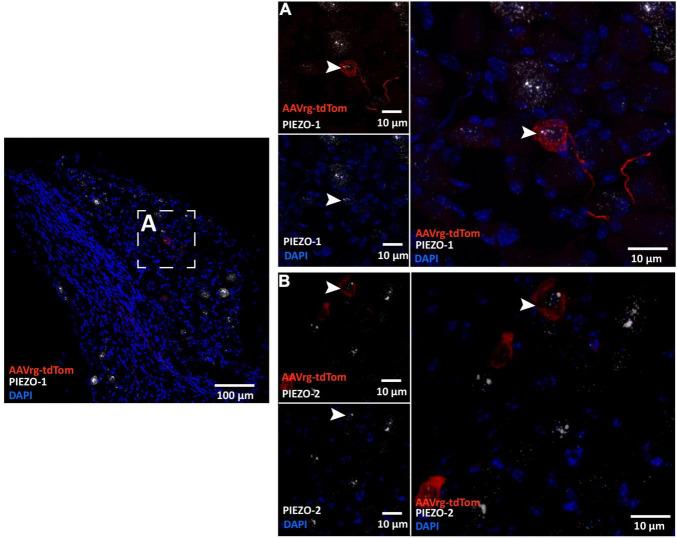
Sensory neurons innervating the aortic arch express the mechanically activated ion channels PIEZO-1 and PIEZO-2. Representative photomicrographs demonstrating the colocalization of the nodose ganglia neurons that expressed tdTom with the mRNAs of **(A)** PIEZO-1 and **(B)** and PIEZO-2.

Cell bodies of sensory neurons innervating cardio-pulmonary and gastrointestinal organs are found within the NDG (Kupari et al., 2019). Recently, mechanically activated ion channels PIEZO-1 and PIEZO-2 have been shown to be expressed in sensory neurons that innervate the aortic arch and sense stretch in the arterial wall (Zeng et al., 2018; Min et al., 2019). Thus, we performed RNAscope *in situ* hybridization to label the mRNAs coding for PIEZO-1 and PIEZO-2 in the NDG of mice that received AAVrg-tdTom into the aortic arch. As expected, we found the cell bodies of the NDG that expressed tdTom to also express both PIEZO-1 (Figure 4A) and PIEZO-2 (Figure 4B). These results demonstrate that our approach is selective in labeling sensory vagal afferents that sense stretch at the level of the aortic arch.

### Fibers of the Sensory Neurons Innervating the Aortic Arch Form Functional Connections With Nucleus of the Solitary Tract Neurons

**FIGURE 5:**
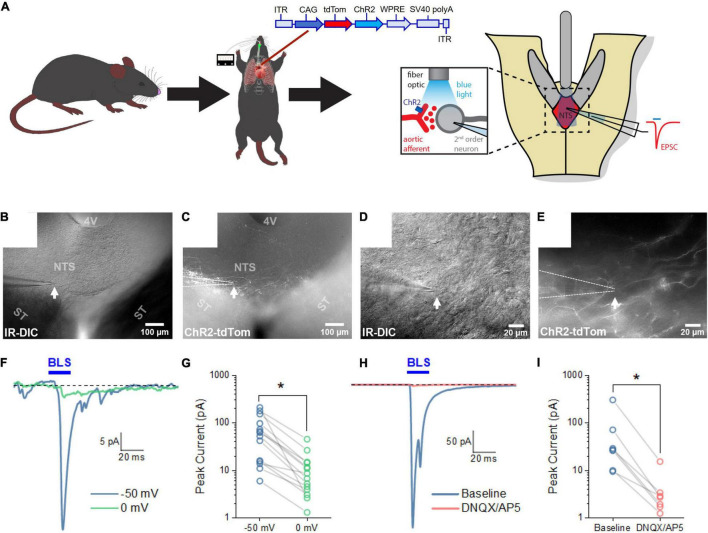
Blue light stimulation of baroreceptors induces glutamate release onto NTS neurons. **(A)** AAVrg applied to the aortic arch to direct the expression of ChR2 and tdTom within sensory neurons innervating the aortic arch (*n* = 7). Light-sensitive synapses in the NTS were characterized using whole-cell patch-clamp in horizontal brain slices. **(B–E)** Neurons in close approximation with ChR2/tdTom axons were targeted for study using a combination of infrared differential interference contrast (IR-DIC) and epifluorescence microscopy. **(F)** Representative traces from an NTS neuron demonstrating an excitatory current produced by 20 ms blue light stimulation (BLS). Light-evoked currents were substantially reduced when neurons were voltage clamped at 0 mV. Traces shown are the mean of 10 consecutive sweeps. **(G)** Mean peak light-evoked inward current at -50 and 0 mV (*n* = 14 cells from seven animals). **(H)** Representative traces demonstrating light-evoked inward current is also strongly attenuated by bath application of glutamate receptor antagonists. Traces shown are the mean of 30 consecutive sweeps. **(I)** Light-evoked currents were significantly reduced by ionotropic glutamate receptor antagonist (*n* = 7).

Next, we next sought to characterize the connectivity between sensory neurons innervating the aortic arch and the NTS. Mice received an AAVrg to direct the expression of ChR2 and tdTom (*n* = 7) within the sensory afferents of the aortic arch. Following recovery, horizontal sections of the NTS were prepared to perform *in vitro* electrophysiology (Figure 5A). NTS neurons in close proximity to fibers expressing ChR2/tdTomato were targeted for whole-cell patch-clamp recording (Figures 5B–E). Following establishment of a whole-cell patch-clamp recording, passive membrane properties were evaluated in voltage clamp at –70 mV using a cesium-based internal solution (see Section “Materials and Methods”). Under these conditions holding current was –13.77 ± 2.78 pA, membrane resistance was 1.23 ± 0.166 GΩ, and whole-cell capacitance was 31.83 ± 2.71 pF (*n* = 14 cells across 7 mice). To evaluate whether these NTS neurons received synaptic inputs from ChR2-expressing axons of baroreceptor expressing neurons, cells were voltage clamped at –50 mV in the continuous presence of the GABA_A_ receptor antagonist PTX (100 μM). Blue light pulses (20 ms) were then used to evoke neurotransmitter release from ChR2/tdTomato-expressing axon terminals arriving from the aortic arch. We found that blue light evoked an excitatory current in 14 of 109 NTS cells tested (–71.41 ± 17.68 pA at –50 mV, *n* = 14 cells across 7 mice, Figures 5E,F). In all cases, light-evoked responses were observed as an inward current at –50 mV, which was significantly reduced at 0 mV (–11.51 ± 3.13 pA, *p* = 4.58e-6, Figures 5F,G). These results indicate that baroreceptor expressing afferents that make synaptic connections with NTS neurons are likely glutamatergic. To further test this hypothesis, a subset of light-evoked responses was subsequently challenged with bath applied glutamate receptor antagonists. Bath-application of ionotropic glutamate receptor antagonists DNQX or DNQX + AP5 effectively eliminated light-evoked currents (amplitude reduced from –68.20 ± 39.97 pA to –4.18 ± 1.90 pA, Figures 5H,I, *p* = 9.31e-5, *n* = 7, 4), confirming they were mediated by activation of glutamatergic receptors. These results demonstrate that the labeled sensory neurons innervating the aortic arch form functional glutamatergic synaptic connections with NTS neurons.

### *In vivo* Optogenetic Excitation of Sensory Neurons Innervating the Aortic Arch Produces Depressor Responses

**FIGURE 6:**
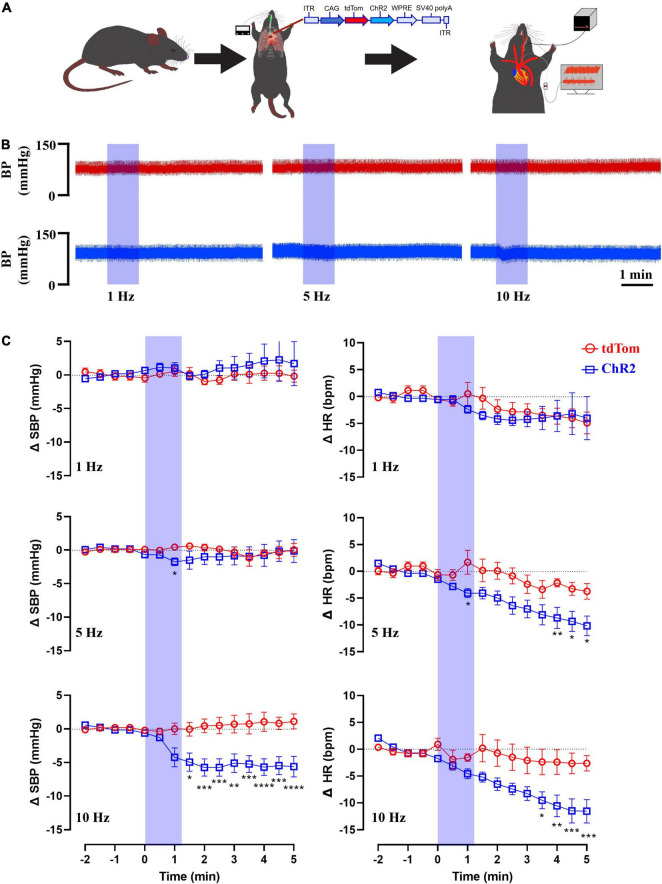
Blue light stimulation of baroreceptors elicits depressor responses. **(A)** AAVrg applied to the aortic arch to direct the expression of ChR2 and/or tdTom within baroreceptors (*n* = 6 per group). **(B)** Representative traces of arterial pressure and heart rate following the optogenetic stimulation of baroreceptors cell bodies at 1, 5, and 10 Hz (473 nm, 10 mW output; 20 ms pulse width; for 1 min). **(C)** Time-course changes in systolic blood pressure (SBP) and heart rate (HR) in control (tdTom) and experimental (ChR2) groups. *p < 0.05, **p < 0.01, ***p < 0.001, ****p < 0.0001; Two-way ANOVA followed by Tukey’s *post hoc* test. *n* = 6 per group.

Next, we assessed the effects of the *in vivo* optogenetic stimulation of the sensory neurons innervating the aortic arch on BP and HR. Mice received an injection of AAVrg to direct the expression of ChR2 and/or tdTom (*n* = 6 per group) within the baroreceptor expressing afferents. Following recovery, the left common carotid artery was catheterized to record BP, and a fiberoptic post was placed above the LNG to optogenetically stimulate the cell bodies of the baroreceptors (Figure 6A). Following baseline recording (Table 1), the optogenetic stimulation (473 nm, 10 mW output; 20 ms pulse width; 1, 5, 10 Hz for 1 min) of the cell bodies of baroreceptor expressing afferents elicited frequency-dependent depressor responses (Figure 6B). In comparison to control groups that only expressed tdTom, BP (tdTom vs. ChR2; 1 Hz: +0.5 ± 0.8 vs. +1.0 ± 0.8 mmHg, 5 Hz: +0.5 ± 0.4 vs. –1.8 ± 0.6 mmHg, 10 Hz: +0.05 ± 0.9 vs. –4.9 ± 1.3 mmHg at 1 min, *n* = 6 per group, Figure 6C) and HR (tdTom vs. ChR2; 1 Hz: +0.5 ± 2.1 vs. –2.4 ± 0.7 bpm, 5 Hz: +1.7 ± 2.2 vs. –4.1 ± 0.8 bpm, 10 Hz: –1.6 ± 0.7 vs. –4.6 ± 0.9 bpm at 1 min, *n* = 6 per group, Figure 6C) were significantly reduced in response to blue light illumination. Altogether, our anatomical and functional data suggest that the current approach targets neurons that express baroreceptors in the aortic arch with high specificity, and further demonstrates selective optogenetic activation of these neurons elicits a depressor response.

**TABLE 1:** Body weights, baseline mean blood pressure (MBP), and heart rate (HR) prior to optogenetic stimulation.

	Body weight (g)	MBP (mmHg)	HR (bpm)
tdTom	29.1 ± 0.5	1 Hz: 75.2 ± 5.9	1 Hz: 487 ± 12
		5 Hz: 77.2 ± 4.4	5 Hz: 473 ± 11
		10 Hz: 77.5 ± 3.6	10 Hz: 472 ± 7
ChR2	29.8 ± 0.9	1 Hz: 80.6 ± 7.6	1 Hz: 449 ± 4
		5 Hz: 78.4 ± 6.1	5 Hz: 462 ± 11
		10 Hz: 78.7 ± 6.7	10 Hz: 465 ± 13

*Data expressed as mean ± SEM, n = 6 per group.*

## Discussion

Sensory nerve endings located at the aortic arch employ baroreceptors that convert stretch exerted on the arterial wall into action potentials carried by the vagus nerve to second order neurons residing within the NTS. This provides moment-to-moment feedback control of HR and BP (Benarroch, 2008; Wehrwein and Joyner, 2013). Here, we used a novel organ-specific approach to selectively target baroreceptor expressing afferents with an AAVrg conjugated to tdTom and/or ChR2. Our anatomical results revealed robust tdTom expression in nerve endings surrounding the aortic arch, within the fibers of the ADN and vagus nerves, cell bodies of the NDG, and neural projections to the cNTS. Additionally, the tdTom labeled cell bodies within the NDG expressed mRNAs coding for the mechanically gated ion channels, PIEZO-1 and PIEZO-2. *In vitro* electrophysiology revealed that pulses of blue light evoked excitatory post-synaptic currents in a subset of neurons within the cNTS, suggesting a functional connection between the labeled aortic arch sensory afferents and second order neurons. Finally, *in vivo* optogenetic stimulation of the cell bodies of baroreceptor expressing afferents in NDG produced robust depressor responses. Altogether, our results demonstrate that the current approach selectively targets and labels baroreceptor expressing afferents in the aortic arch.

Viscero-sensory afferents convey the status of visceral organs to second order neurons in the NTS (Andresen and Paton, 2011). This includes physiological signals from cardio-pulmonary and gastrointestinal tract (GIT) organs that project to the NTS through the vagus nerve, where the cell bodies of these afferents reside in the NDG (Berthoud and Neuhuber, 2000; Kubin et al., 2006; Bai et al., 2019; Kupari et al., 2019). The recent development of advanced genetic tools allows for the anatomical and functional characterization of vagal sensory afferents with spatial and temporal resolution (Saleeba et al., 2019). Using the Cre-LoxP system and virally mediated gene transfer targeted to the GIT, it was possible to anatomically and functionally characterize vagal sensory neurons that innervate the GIT (Han et al., 2018). While these advanced genetic technologies allowed for the selective characterization of sensory circuits, viscero-sensory afferents, and neural reflexes, baroreceptor expressing sensory afferents are yet to be fully characterized. This is due to the fact that selective targeting of such afferents is challenging. Unlike the GIT, where viral vectors can be injected into the gut wall, injection of viral vectors into the vasculature is problematic. Furthermore, the aortic arch is located in the thoracic cavity which is difficult to access in small rodents for recovery surgeries. To circumvent these challenges, we have developed a novel approach that selectively targets and labels sensory neurons innervating the aortic arch. This approach involves (1) a novel surgical approach that exposes the aortic arch in mice, and (2) the application of viral vectors to the aortic arch by “painting” the virus on the vascular wall.

Our anatomical studies demonstrate the selective targeting of fibers that convey a single modality to the NTS. Following the application of an AAVrg to the aortic arch, we directed the expression of tdTom within sensory afferents that contain baroreceptors. Consistent with previous studies describing the anatomy/morphology of baroreceptor containing afferents (Aumonier, 1972; Krauhs, 1979; Min et al., 2019), we found abundant tdTom expression within nerve endings surrounding the aortic arch. The morphology of these terminals resembled flower-spray endings, spanning the dorsal and ventral surface of the aortic arch in a claw-like pattern. Both dorsal and ventral fibers joined the ADN and terminated in the cNTS through the vagus nerve. This is consistent with previous studies that anatomically mapped the projections of vagal baroreceptors (Cottle, 1964; Lipski et al., 1975). For example, following the selective transection of the vagus nerve in cats, axonal and terminal degeneration were found in the NTS (Cottle, 1964). Furthermore, antidromic stimulation of baroreceptor expressing fibers, demonstrated that these fibers terminate in the NTS (Lipski et al., 1975). In addition, previous studies demonstrated that cardiovascular vagal afferents terminate in the cNTS (Mendelowitz et al., 1992), whereas afferents arising from the GIT terminate in the rostral NTS (Han et al., 2018). This suggests that our approach selectively targets aortic arch afferents that terminate in the caudal portion of the NTS.

Sensory neurons innervating the aortic arch convey chemosensory and mechanosensory signals to the NTS. It is widely accepted in the literature that fibers in the ADN contain baroreceptors. Electrical stimulation of the ADN produces robust depressor responses (Schmidt, 1968; Sapru et al., 1981; Fan et al., 1996). Furthermore, electrophysiological recordings from the ADN demonstrate a change in discharge to hemodynamic stimuli (Kobayashi et al., 1999). However, studies utilizing recordings from single ADN fibers demonstrate that some fibers change their firing in response to switching from normocapnic hyperoxia and hypercapnic hypoxia (Brophy et al., 1999). These results suggest that fibers in the ADN sense both chemosensory and mechanosensory stimuli. More recently, baroreceptors sensing stretch (mechanosensory) at the aortic arch were shown to express the mechanically gated ion channels, PIEZO-1 and PIEZO-2 (Zeng et al., 2018; Min et al., 2019). Therefore, to examine whether our approach specifically labels baroreceptors, we used RNAscope *in situ* hybridization to label the mRNAs coding for both PIEZOs in the NDG. We found the labeled NDG neurons that received input from the aortic arch express both PIEZO-1 and PIEZO-2. This suggests that the current approach labels sensory afferents that utilize mechanically gated ion channels to detect stretch at the aortic arch. However, it is possible that the current approach also labels chemosensory afferents. Previous studies demonstrate that the majority of chemoreceptor expressing afferents terminate in the most caudal portion of the NTS (Colombari and Talman, 1995; Colombari et al., 1996), whereas baroreceptor expressing afferents terminate in the caudal and intermediate portions of the NTS (Mendelowitz et al., 1992). Our confocal scans of the entire brainstem revealed that labeled fibers from the aortic arch were only found in the caudal and intermediate NTS. Moreover, nerve fibers terminating at the aortic arch exhibited distinct baroreceptor morphology, characterized by abundant flower-spray endings (Cheng et al., 1997; Min et al., 2019). These labeled fibers did not exhibit chemoreceptor morphology which is described as pericellular endings innervating small intensity fluorescent cell bodies. These chemosensory nerve endings are abundantly found at the carotid sinus (Cheng et al., 1997) and we obtained additional confocal scans of the carotid sinus to discern whether our approach also labeled chemosensory afferents. We did not observe any nerve endings or fibers at the carotid sinus (Supplementary Figure 1); however, the same animals exhibited robust and apparent tdTom labeled fibers at the aortic arch (Figure 1). This suggests that our approach is selective in labeling mechano-sensory afferents at the aortic arch that presumably contain baroreceptors. Thus, our current approach may be utilized to genetically manipulate baroreceptor expressing sensory afferents to examine their physiological function.

Baroreceptors sense stretch at the wall of the aortic arch and activate the baroreflex to modulate BP at homeostatic levels. An increase in BP also increases stretch on the wall of the aortic arch, stimulating baroreceptors (Kirchheim, 1976). This activates the baroreceptors to trigger afferent signals conveyed to the NTS (Benarroch, 2008; Wehrwein and Joyner, 2013). Subsequently, stimulating a neural reflex (known as the baroreflex) that reduces HR by reducing sympathetic outflow and increasing parasympathetic output (Zeng et al., 2018; Min et al., 2019). This reduces BP back to homeostatic levels (Spyer, 1989; Andresen and Kunze, 1994). Conversely, a drop in BP (such as in postural hypotension) triggers baroreceptor unloading leading to increases in BP (Thrasher, 2005). Thus, baroreceptors detect moment-to-moment fluctuations in BP to maintain vascular resistance and cardiovascular output at optimal levels, ensuring sufficient blood flow to the body. Here, we used an AAVrg to direct the expression of ChR2 within nerve endings surrounding the aortic arch to stimulate baroreceptors. Our *in vitro* electrophysiological studies confirmed the functionality of the ChR2 molecule in baroreceptor expressing afferents, as pulsing blue light on ChR2 expressing fibers in the cNTS elicited glutamatergic-dependent excitatory post-synaptic currents in NTS neurons. Thus, we utilized an *in vivo* approach to record BP while the cell bodies of afferents containing baroreceptors in the NDG were optogenetically activated to simulate mechano-stimulation. Consistent with previous studies demonstrating that the electrical stimulation of the ADN results in hypotension (Schmidt, 1968; Sapru et al., 1981; Fan et al., 1996), we observed robust reductions in BP and HR upon blue light stimulation of baroreceptor containing neurons. This suggests that our current approach can be combined with advanced genetic technologies to selectively interrogate the physiological function of baroreceptor expressing vagal afferents.

The results of the current study demonstrate that our approach is highly selective in targeting baroreceptor expressing sensory afferents. This warrants future studies that interrogate pathophysiological function of these afferents. In hypertension, 19% of patients suffer from drug-resistant hypertension despite the plethora of anti-hypertensive treatments (Carey et al., 2019). This is often termed as “neurogenic hypertension” due to the neuronal impairments that lead to augmented sympathetic outflow (Smith et al., 2004, 2002; Oliveira-Sales et al., 2016; Korim et al., 2019). Some of the first evidence for the role of baroreceptor fibers in cardiovascular control and hypertension, originated from denervation studies (Krieger, 1964). In this study, 140 rats were subjected to denervation of aortic depressor fibers, and were observed for one year. Rats exhibited a hypertensive phenotype one week following denervation, where the hypertension was permanent in 75% of the rats observed up to one year (Krieger, 1964). This study demonstrates that integrity of baroreceptor expressing afferents is crucial for maintaining BP. In fact, baroreflex dysfunction is well documented in hypertension (Parmer et al., 1992; Oliveira-Sales et al., 2016). Baroreflex sensitivity is impaired in hypertension leading to failure in maintaining BP levels (Brook and Julius, 2000). Interestingly, it has been demonstrated that baroreflex dysfunction precedes sympathetic augmentation in rodent models of hypertension, which altogether occurs prior to BP elevations and the establishment of hypertension (Oliveira-Sales et al., 2016). Thus, exploiting the modulatory influence of the afferent baroreceptor signals may have potential therapeutic effects in hypertension.

While the current manuscript evaluates the specificity of a novel approach to express light sensitive molecules in baroreceptor expressing neurons, there are some limitations. The current study utilizes *in vitro* electrophysiology to assess the functionality of the ChR2 molecule. We demonstrate that pulsing light on terminals in the NTS originating from baroreceptor expressing neurons in the NDG reliably produces glutamatergic-dependent excitatory post-synaptic currents in NTS neurons. Further direct validation of the ChR2 molecule used in the current manuscript may require whole-cell recordings of somatic responses to optogenetic stimulation in baroreceptor expressing neurons in the NDG itself. Additionally, the current manuscript does not assess the long-term stability of the ChR2 molecule. This could be achieved by expressing ChR2 in baroreceptor containing neurons for a longer duration than in the current study. This would be followed by assessing the long-term expression of the ChR2 molecule by using *in vitro* electrophysiology and *in situ* hybridization for mRNAs for ChR2. Moreover, *in vivo* optogenetic studies are used to evaluate changes in BP following optogenetic stimulation. In these studies, we found the somatic stimulation of baroreceptor expressing neurons in the NDG to produce depressor responses; however, *in vivo* optogenetic stimulation combined with electrophysiological nerve recording of the ADN and stimulation of fibers in the NTS would more directly validate the functional connectivity between baroreceptor expressing neurons in the NDG and the NTS through the ADN. Finally, the current study uses stimulation frequencies of 1–10 Hz for *in vivo* optogenetic studies. While baroreceptor expressing fibers respond differentially to electrical stimulation (C-type: 1–10 Hz, A-type: >10 Hz) (Fan and Andresen, 1998), the optogenetic somatic stimulation of baroreceptor expressing neurons in the NDG that express ChR2 are expected to achieve suprathreshold depolarization when exposed to pulses of blue light at that location, regardless of whether the axons are unmyelinated or myelinated (Lin et al., 2009).

In conclusion, the present study establishes a novel approach to selectively target single modality vagal afferent fibers that convey stretch at the aortic arch to the NTS. Furthermore, we demonstrate that our approach can be combined with advanced genetic tools to interrogate the physiological function of baroreceptors. Using the current approach may lead to characterizing the molecular, anatomical, functional, and pathological phenotype of baroreceptors with high spatial and temporal resolution.

## Data Availability Statement

The original contributions presented in the study are included in the article/Supplementary Material, further inquiries can be directed to the corresponding authors.

## Ethics Statement

All procedures were reviewed and approved by the Institutional Animal Care and Use Committees at the University of Florida and were conducted in accordance with the National Institutes of Health Guide for the Care and Use of Laboratory Animals.

## Author Contributions

KE, ADK, and EGK conceived, designed, supervised, and coordinated the study, and wrote the manuscript. KE performed the cardiothoracic surgeries. KE, SWH, DNJ, WS, JAS, and KAS conducted experiments and acquired data. KE, AKK, SWH, and CJF analyzed data. KE, CJF, ADK, and EGK contributed unpublished reagents and analytic tools. KE and SWH wrote the first draft of the manuscript. KE, SWH, DNJ, WS, JAS, KAS, CJF, ADK, and EGK edited and revised the manuscript. All authors contributed to the article and approved the submitted version.

## Conflict of Interest

The authors declare that the research was conducted in the absence of any commercial or financial relationships that could be construed as a potential conflict of interest.

## Publisher’s Note

All claims expressed in this article are solely those of the authors and do not necessarily represent those of their affiliated organizations, or those of the publisher, the editors and the reviewers. Any product that may be evaluated in this article, or claim that may be made by its manufacturer, is not guaranteed or endorsed by the publisher.
