# Supplementary material for: A Novel Organ-Specific Approach to Selectively Target Sensory Afferents Innervating the Aortic Arch
Source: Front Physiol. 2022 Mar 24;13:841078. doi: 10.3389/fphys.2022.841078 (PMC8987286; doi:10.3389/fphys.2022.841078)
Supplement: Supplementary file 1 [file Image_1.pdf]

Supplementary Material

1 Supplementary Figures

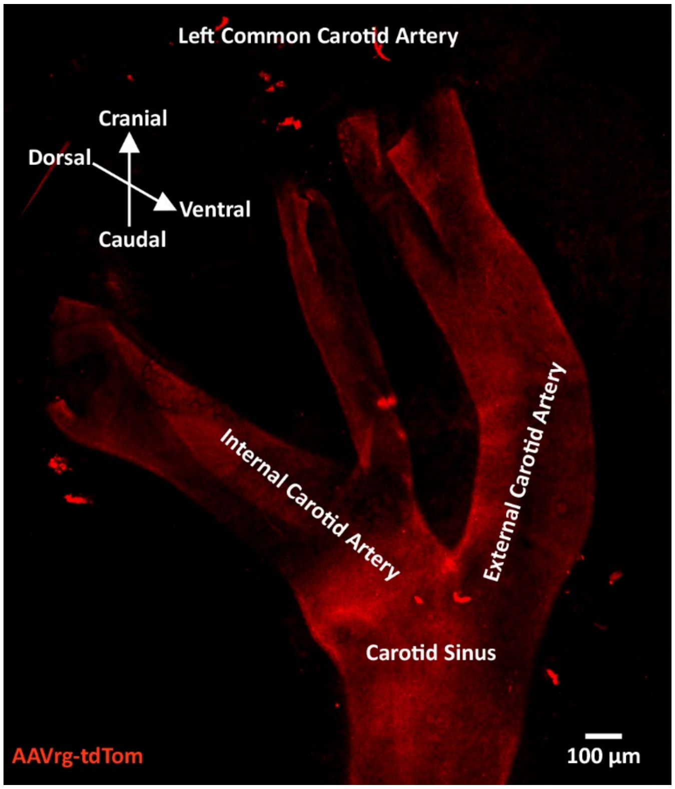

Supplementary Figure 1. A representative photomicrograph of a whole-mount left common carotid artery from the animal that received an AAVrg to direct the expression of tdTom within the sensory neurons innervating the aortic arch in Figure 1. This figure demonstrates that the application of viral construct to the aortic arch is contained within the arch and does not circulate to or transfect the carotid bifurcation.
